# Effectiveness and Safety of Iguratimod in Treating Primary Sjögren’s Syndrome: A Systematic Review and Meta-Analysis

**DOI:** 10.3389/fphar.2021.621208

**Published:** 2021-03-19

**Authors:** Jincheng Pu, Xuan Wang, Farooq Riaz, Tongyangzi Zhang, Ronglin Gao, Shengnan Pan, Zhenzhen Wu, Yuanyuan Liang, Shuqi Zhuang, Jianping Tang

**Affiliations:** ^1^ Department of Rheumatology and Immunology, Tongji Hospital, Tongji University School of Medicine, Shanghai, China; ^2^ Department of Biochemistry and Molecular Biology, School of Basic Medical Sciences, Xi'an Jiaotong University Health Science Center, Xi’an, China

**Keywords:** iguratimod, primary Sjögren’s syndrome, effectiveness, safety, meta-analysis

## Abstract

**Objectives:** We aimed to assess the effectiveness and safety of iguratimod (IGU) in treating primary Sjögren’s syndrome (pSS) by meta-analysis.

**Methods:** Eight databases and two clinical trial websites were searched from conception to August 10, 2020, for relevant randomized controlled trials (RCTs) on outcomes of patients with pSS treated with IGU. Revman 5.4 was used for statistical analysis and creating plots.

**Results:** A total of 1,384 patients with pSS from 19 RCTs were included in this meta-analysis. Pooled results demonstrated that patients treated with IGU + hydroxychloroquine (HCQ) + glucocorticoid (GC) showed significant differences in erythrocyte sedimentation rate (ESR), rheumatoid factor (RF) level, platelet (PLT) count, immunoglobulin G (IgG) level, salivary flow rate, Schirmer’s test result, EULAR Sjögren’s Syndrome Patient Reported Index (ESSPRI), EULAR Sjögren’s Syndrome Disease Activity Index (ESSDAI), and efficacy rate (*p* ≤ 0.01) compared to patients treated with HCQ + GC. Compared to treatment with HCQ and GC, co-administration of IGU with GC showed significant differences in ESR and RF level (*p* ≤ 0.01); however, no significant differences were noted in IgG level. Conversely, the IgG level showed a significant improvement in the IGU + HCQ + GC group compared to the HCQ + GC group. The results of safety analysis revealed that seven trials showed no significant differences in adverse events (AEs) between the IGU + HCQ + GC and HCQ + GC groups (*p* = 0.15). Although no severe AEs were noted, gastrointestinal discomfort was the most common AE in the IGU group. No significant differences in AEs were observed between the IGU + GC and HCQ + GC groups.

**Conclusion:** IGU improved the clinical symptoms of patients with pSS, including inflammatory indicators (ESR, IgG, and RF levels), PLT count, secretion function of the salivary and lacrimal glands (salivary flow rate and Schirmer’s test result), and disease indexes (ESSDAI and ESSPRI), when co-administered with HCQ + GC therapy without increasing the risks of AEs. Therefore, IGU can be considered as an effective and safe drug for clinical therapy of pSS. Considering the limitations of the present trials, more long-term, multicenter, and high-quality RCTs are required to assess the effectiveness and safety of IGU for treating patients with pSS.

## Introduction

Primary Sjögren’s syndrome (pSS) is an autoimmune disease related to the dysfunction of exocrine glands caused by lymphocytic infiltration along with xerostomia and xerophtalmia (Nair and Singh, 2017; Bowman, 2018). Prior studies have concluded that pSS is 10 times more likely to occur in females than in males and could develop at any age with an average onset age of 50 years (Nair and Singh, 2017). Recent epidemiological data showed that the prevalence of pSS in China is approximately 0.29–0.77% (Chinese Rheumatology Association, 2010), whereas the worldwide incidence and prevalence rates of pSS are 6.92 (95% CI: 4.98–8.86) per 100,000 persons and 60.82 (95% CI: 43.69–77.94) per 100,000 persons, respectively (Qin et al., 2015). The main symptoms such as impaired glandular function, fatigue, and musculoskeletal pain cause long-term severe physical limitations, severe complications, massive psychological pressure, and large financial burdens on patients with pSS (Bowman, 2018). This huge impact on patients’ quality of life has made pSS a hot topic of research for scientists worldwide.

As the pathogenic mechanisms of pSS remain unclear, specific targeted drugs are yet to be discovered. Generally, the appropriate clinical treatment regimen of pSS is determined after assessing the overall symptoms of the patients and the involvement of an organ or system. Immunosuppressant/disease-modifying anti-rheumatic drugs (DMARDs), including hydroxychloroquine (HCQ), prednisone, methotrexate, mycophenolate sodium, azathioprine, and cyclosporine, are still considered the most important drugs in the remission of extra-glandular symptoms (Vivino et al., 2019). However, their non-specificity and the associated adverse events (AEs) should be considered. In the past few years, several biological agents have been developed and used for treating pSS (Fasano and Isenberg, 2019). However, more randomized controlled trials (RCTs) are needed to prove the effectiveness of these biological agents. Moreover, these biological agents should be available at more affordable prices, which seems impossible in the short term. Therefore, more research is needed to find a safer, cheaper, and more effective treatment for pSS.

Iguratimod (IGU), a small-molecule compound that is being widely used in China and Japan, is a potential anti-rheumatic drug and is used for the treatment of several rheumatic diseases (Jiang H. et al., 2020). An advantage of IGU is its inhibition of the functions of B cells by reducing the production of immunoglobulin and various inflammatory cytokines, including interleukin (IL)-1, IL-6, IL-8, and tumor necrosis factor (TNF) (Xu et al., 2015). Several research studies have documented IGU as an effective therapeutic agent for pulmonary fibrosis and osteoporosis (Wu et al., 2017). The multi-effects of IGU on immune modulation has made it a widely used drug for treating rheumatic diseases, including pSS. Although several studies have been conducted to assess the efficacy of IGU for treating pSS (Jiang et al., 2014; Jiang et al., 2016; Xia et al., 2017; Xu et al., 2017; Li et al., 2018; Liao, 2018; Luo et al., 2018; Wang, 2018; Bai and Jiao, 2019; Ji and Cheng, 2019; Wang et al., 2019; Zhang, 2019; Zhang and Shen, 2019;Zhao, 2019; Jiang W. et al., 2020; Li and Long, 2020; Lin and Zhang, 2020; Wang and Wang, 2020; Yu, 2020), no consensus has been reached. Here, we aimed to analyze the effectiveness and safety of IGU for treating pSS by performing a comprehensive meta-analysis of the available data.

## Methods

The Preferred Reporting Item for Systematic Reviews and Meta-Analyses (PRISMA) Statement (Moher et al., 2009) and protocol registration in PROSPERO (CRD42020204889) were strictly observed for this review.

### Literature Search

Eight databases, namely PubMed, EBSCOhost, Cochrane Central Register of Controlled Trials (CENTRAL), embase, Chinese Biomedical (CBM) database, China National Knowledge Infrastructure (CNKI), Chinese VIP Information database, and Wanfang Med Database, and two clinical trial websites, namely Clinicaltrials and Chinese Clinical Trial Registry, were searched from their conception to August 10, 2020 by two authors independently. Only trials published in the English and Chinese languages were searched. The following search terms were used in the first four English databases: iguratimod OR IGU OR elamode OR T-614 AND Sjogrens Syndrome OR Sjögren’s Syndrome OR syndrome, Sjögren’s OR Sjogren’s Syndrome OR syndrome, Sjogren’s OR Sjogren Syndrome OR Sjoegren Syndrome OR SS OR SjS OR Syndrome, Sicca OR Sicca Syndrome. Search terms such as “ai la mo de” (the Chinese name of iguratimod) and “gan zao zong he zheng” (the Chinese name of Sjögren's syndrome) were used in the other four Chinese databases.

### Screening of Literature

#### Inclusion Criteria

1. (P) Patients diagnosed with pSS following the international classification in 2002 or 2016 American College of Rheumatology (ACR)-European League Against Rheumatism (EULAR) classification criteria for pSS (Vitali et al., 2002; Shiboski et al., 2016);

2.  (I&C) Patients who had taken IGU and other immunosuppressors (ISs) in the experimental group, but other ISs without IGU in the control group;

3. (O) The outcomes included erythrocyte sedimentation rate (ESR), rheumatoid factor (RF) level, immunoglobulin G (IgG) level, platelet (PLT) count, salivary flow rate, Schirmer’s test result, EULAR Sjögren’s Syndrome Disease Activity Index (ESSDAI), EULAR Sjögren’s Syndrome Patient Reported Index (ESSPRI), efficacy rates, and AEs;

4. Only RCTs investigating the effectiveness and safety of IGU in treating pSS were included in this review. Dissertations were also considered for inclusion.

#### Exclusion Criteria

1.  Research studies with incomplete, repetitive, or unextractable data;

2.  Studies focusing on a specific population;

3.  Review articles, case reports, meeting minutes, commentaries, or other incomplete published research studies;

4.  Studies whose data were incomplete or unclear;

5.  Studies of before-after, non-RCTs, and conference abstracts.

### Data Extraction and Analysis

On the basis of the previously mentioned selection criteria, the related data, including author’s name, publication year, study design, characteristics of participants, interventions of each group, treatment duration, outcomes, and AEs, were rigorously extracted by two authors (JCP and RLG) independently, and verified by the third author (XW). Later, the two authors (JCP and RLG) used the Cochrane risk of bias tool (Higgins and Green, 2011) to assess the methodological quality of each included study independently. The risk of bias comprised random sequence generation (selection bias), allocation concealment (selection bias), blinding of participants and personnel (performance bias), blinding of outcome assessment (detection bias), incomplete outcome data (attrition bias), selective reporting (reporting bias), and other biases in this tool, and was classified as “low risk,” “unclear risk,” and “high risk” for each term. In the case of any disagreement, the first and second authors discussed initially and then consulted the third author (XW) for a final decision.

### Statistical Analysis

We mainly used Revman 5.4 in this meta-analysis. The odds ratio (OR) was used for dichotomous data, and mean difference (MD) or standardized mean difference (SMD) and 95% CI were used for continuous data. To assess statistical heterogeneity, the I-squared and chi-squared tests were used. For *p* ≥ 0.1 in the chi-squared test or *I*-squared ≤ 50%, a fixed-effect model was used; otherwise, a random-effect model was applied. In this review, statistical significance was considered at the 5% level. For highly heterogeneous data, subgroup analysis and sensitivity analysis were performed.

## Results

### Characteristics of the Included Trials

**FIGURE 1:**
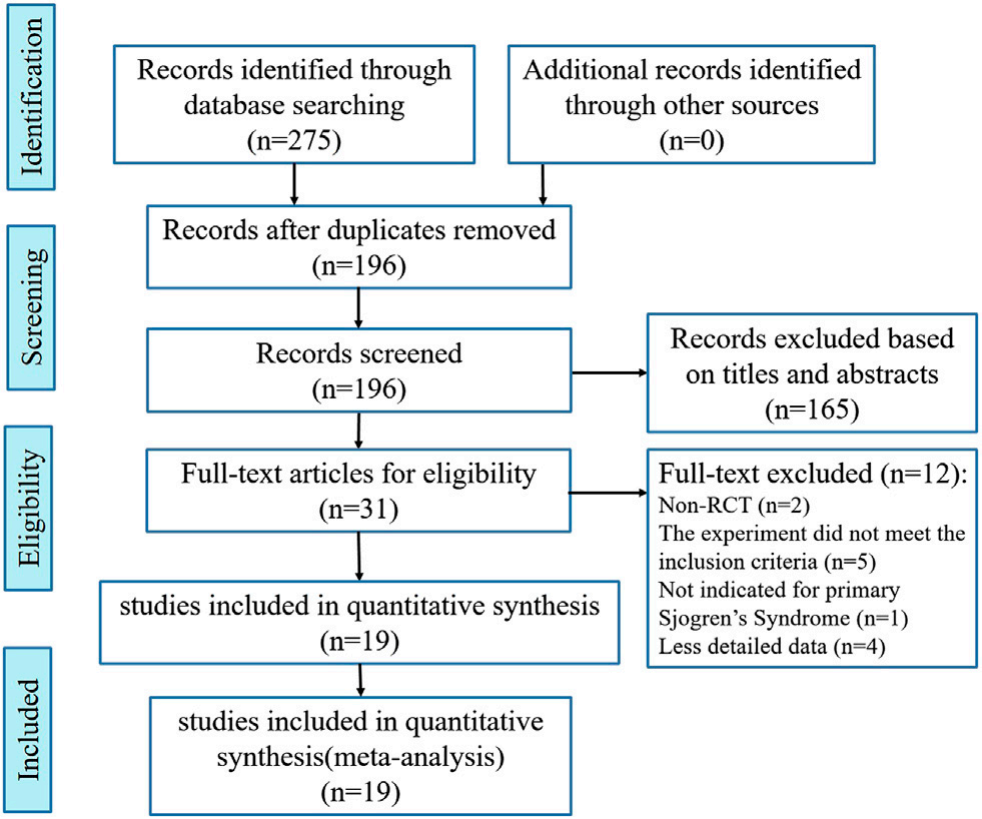
Flow chart of trial selection.

Following the search strategy, 275 records were retrieved; of these records, 79 records were excluded because of duplication and 165 records were excluded after screening their titles and abstracts. Finally, 19 RCTs were included in the meta-analysis, and their full-text articles were further analyzed. These RCTs included a total of 1,384 participants grouped into the experimental group or the control group (IGU + HCQ + GC vs. HCQ + GC (n = 672) (Jiang et al., 2014; Jiang et al., 2016; Xu et al., 2017; Li et al., 2018; Wang, 2018; Ji and Cheng, 2019; Zhang and Shen, 2019; Jiang W. et al., 2020; Li and Long, 2020; Wang and Wang, 2020); IGU + GC vs. HCQ + GC (n = 548) (Xia et al., 2017; Luo et al., 2018; Zhang, 2019; Zhao, 2019; Lin and Zhang, 2020; Yu, 2020); IGU + HCQ + TGP vs. HCQ + TGP (n = 104) (Liao, 2018; Wang et al., 2019); IGU + HCQ + GC vs. LEF + HCQ + GC (n = 60) (Bai and Jiao, 2019)). The workflow of literature screening is presented in Figure 1, and the characteristics of the final 19 included studies are shown in Table 1.

**TABLE 1:** The characteristics of included studies.

Author	Year	Study design	Gender (M/F)	Age (year)	Sample size		Intervention and dose		Treatment duration	Main outcomes	Reference
					Experimental	Control	Experimental	Control			
Jiang W	2014	Prospective	0/50	29.3 ± 9.7 32.5 ± 11.5	25	25	IGU 25 mg bid + HCQ 200 mg bid + P 5–10 mg qd	HCQ 200 mg bid + P 5–10 mg qd	12w	PLT, IgG, Schirmer’s test, ESSPRI, ESSDAI, effective rate, AEs	Jiang et al. (2014)
Jiang DX	2016	Prospective	0/60	28–76	30	30	IGU 25 mg bid + HCQ 200 mg bid + MP 8 mg qd	HCQ 200 mg bid + MP 8 mg qd	12w	ESR, RF, IgG, AEs	Jiang et al. (2016)
Xia ZB	2017	Prospective	0/100	24–76	50	50	IGU 25 mg bid + MP 8 mg qd	HCQ 200 mg bid + MP 8 mg qd	12w	ESR, RF, IgG	Xia et al. (2017)
Xu D	2017	Prospective	12/82	28–77	47	47	IGU 25 mg bid + HCQ 200 mg bid + MP 8 mg qd	HCQ 200 mg bid + MP 8 mg qd	3 m	ESR, RF, PLT, IgG, Schirmer's test, Salivary flow rate, ESSPRI, ESSDAI, effective rate, AEs	Xu et al. (2017)
Li CJ	2018	Prospective	29/39	NF	34	34	IGU 25 mg bid + HCQ 200 mg bid + MP 8 mg qd	HCQ 200 mg bid + MP 8 mg qd	12w	ESR, RF, IgG, Salivary flow rate, ESSPRI, effective rate, AEs	Li et al. (2018)
Luo QW	2018	Prospective	9/71	30–76	40	40	IGU 25 mg bid + MP 8 mg qd	HCQ 200 mg bid + MP 8 mg qd	3 m	Effective rate, AEs	Luo et al. (2018)
Liao Y	2018	Prospective	0/40	25–69	20	20	IGU 25 mg qd-bid + HCQ 0.2 g bid + TGP 0.6 g tid	HCQ 0.2 g bid + TGP 0.6 g tid	12w	ESR, IgG	Liao (2018)
Wang Y	2018	Retrospective	13/63	27–78	38	38	IGU 25 mg bid + HCQ 0.2 g bid + MP 8 mg qd	HCQ 0.2 g bid + MP 8 mg qd	12w	ESR, RF, IgG, effective rate	Wang (2018)
Bai J	2019	Prospective	NF	25–76	30	30	IGU 25 mg bid + HCQ 0.2 g bid + MP 8 mg qd	LEF 10–50 mg qd + HCQ 0.2 g bid + MP 8 mg qd	12w	RF, IgG, ESR, AEs, ESSDAI, ESSPRI	Bai and Jiao (2019)
Ji JH	2019	Prospective	10/72	30–65	41	41	IGU 25 mg bid + HCQ 0.2 g bid + MP 8 mg qd	HCQ 0.2 g bid + MP 8 mg qd	12w	ESR, RF, PLT, IgG, Schirmer's test, Salivary flow rate, ESSPRI, ESSDAI, effective rate	Ji and Cheng (2019)
Wang YL	2019	Prospective	5/59	60–83	32	32	IGU 25 mg bid + HCQ 0.1 g bid + TGP 0.6 g bid	HCQ 0.1 g bid + TGP 0.6 g bid	12w	ESR, RF, IgG, Schirmer's test, Salivary flow rate, ESSPRI, ESSDAI, effective rate, AEs	Wang et al. (2019)
Zhang J	2019	Prospective	25/61	24–63	43	43	IGU 25 mg bid + HCQ 200 mg bid + MP 8 mg qd	HCQ 200 mg bid + MP 8 mg qd	12w	ESR, RF, PLT, IgG, Schirmer's test, Salivary flow rate, ESSPRI, ESSDAI, effective rate, AEs	Wang et al. (2019)
Zhang XY	2019	Prospective	36/84	28–71	60	60	IGU 25 mg bid + MP 8 mg qd	HCQ 0.2 g bid + MP 8 mg qd	3 m	Schirmer's test, ESSDAI, effective rate	Zhang (2019)
Zhao L	2019	Prospective	47/35	35–75	41	41	IGU 25 mg bid + MP 8 mg qd	HCQ 0.2 g bid + MP 8 mg qd	12w	ESR, RF, IgG, effective rate, AEs	Zhao (2019)
Li RR	2020	Prospective	0/46	23–77	23	23	IGU 25 mg bid + HCQ 0.2 g bid + MP 8 mg qd	HCQ 0.2 g bid + MP 8 mg qd	12w	ESR, IgG, Salivary flow rate, ESSPRI, effective rate, AEs	Li and Long (2020)
Lin T	2020	Prospective	15/75	32–75	45	45	IGU 25 mg bid + MP 8 mg qd	HCQ 0.2 g bid + MP 8 mg qd	12w	Effective rate, AEs	Lin and Zhang (2020)
Jiang W	2020	Prospective	NF	29.3 ± 9.7	25	25	IGU 25 mg bid + HCQ 0.2 g bid + P 10 mg qd	HCQ 0.2 g bid + P 10 mg qd	12w	PLT, IgG, Schirmer's test, ESSPRI, ESSDAI, AEs	Jiang W. et al. (2020)
Wang ZH	2020	Prospective	15/45	32–78	30	30	IGU 25 mg bid + HCQ 0.2–0.4 g bid + MP 8 mg qd	HCQ 0.2–0.4 g bid + MP 8 mg qd	NF	Effective rate	Wang and Wang. (2020)
Yu WJ	2020	Prospective	33/43	20–50	38	38	IGU 25 mg bid + MP 8 mg qd	HCQ 0.2 g bid + MP 8 mg qd	12w	ESR, RF, IgG	Yu (2020)

M, male; F, female; IGU, iguratimod; HCQ, hydroxychloroquine; P, prednisone; MP, methyl prednisolone; TGP, the total glucosides of paeony; NF, not found; ESSPRI, EULAR Sjogren's Syndrome patient reported index; ESSDAI, EULAR Sjögren's Syndrome disease activity index; AEs, adverse event rates; ESR, erythrocyte sedimentation rate; RF, rheumatoid factor; IgG, immunoglobulin G; LEF, leflunomide.

### Quality Assessment of the Included Studies

**FIGURE 2:**
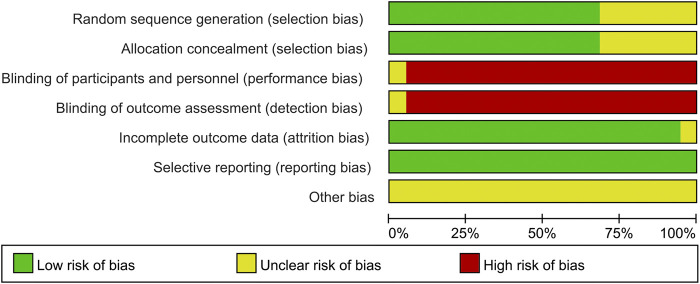
Risk of bias graph of the included studies.

**FIGURE 3:**
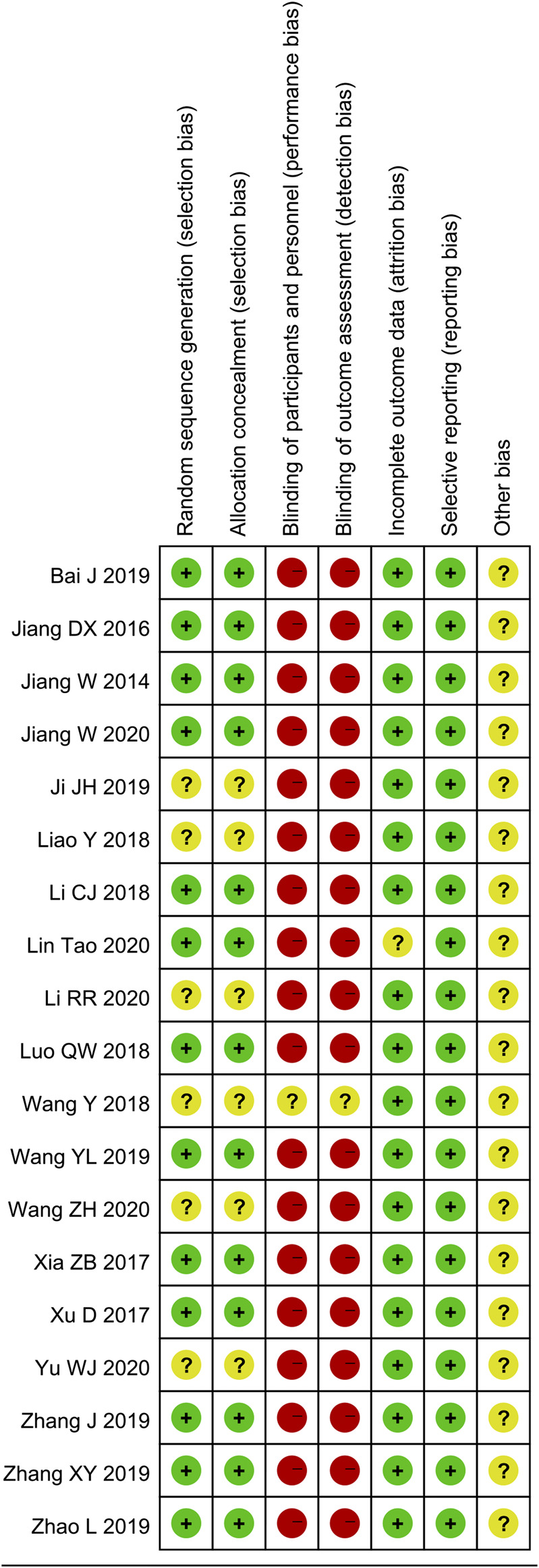
Risk of bias summary of the included studies.

Randomization methods were reported in 13 of the 19 trials (68.4%) and were mentioned without details in the other six trials (31.6%). Allocation concealment was reported in 13 studies (68.4%). Data regarding the blinding of participants, personnel, and outcome assessment were not found in the 19 studies. The complete outcome data were described in 18 studies (94.7%). A low risk of selective reporting and unclear risk of other biases were observed in all studies (100%) (Figures 2 and 3).

### Effects of Interventions

#### IGU + HCQ + GC vs. HCQ + GC

**FIGURE 4:**
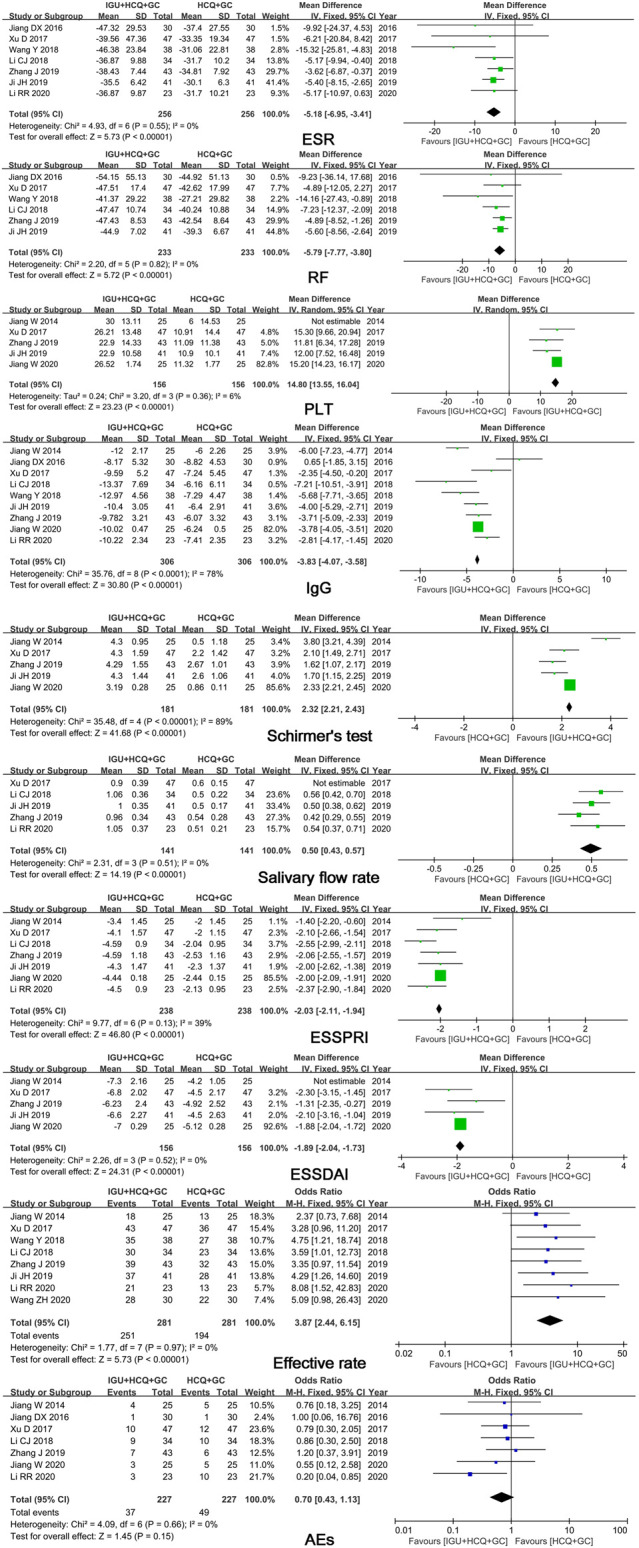
Forest plots of comparison: IGU + HCQ + GC vs. HCQ + GC, outcome: ESR, RF, PLT, IgG, Schirmer’s test, salivary flow rate, ESSPRI, ESSDAI, efficacy rate, AEs.

**FIGURE 5:**
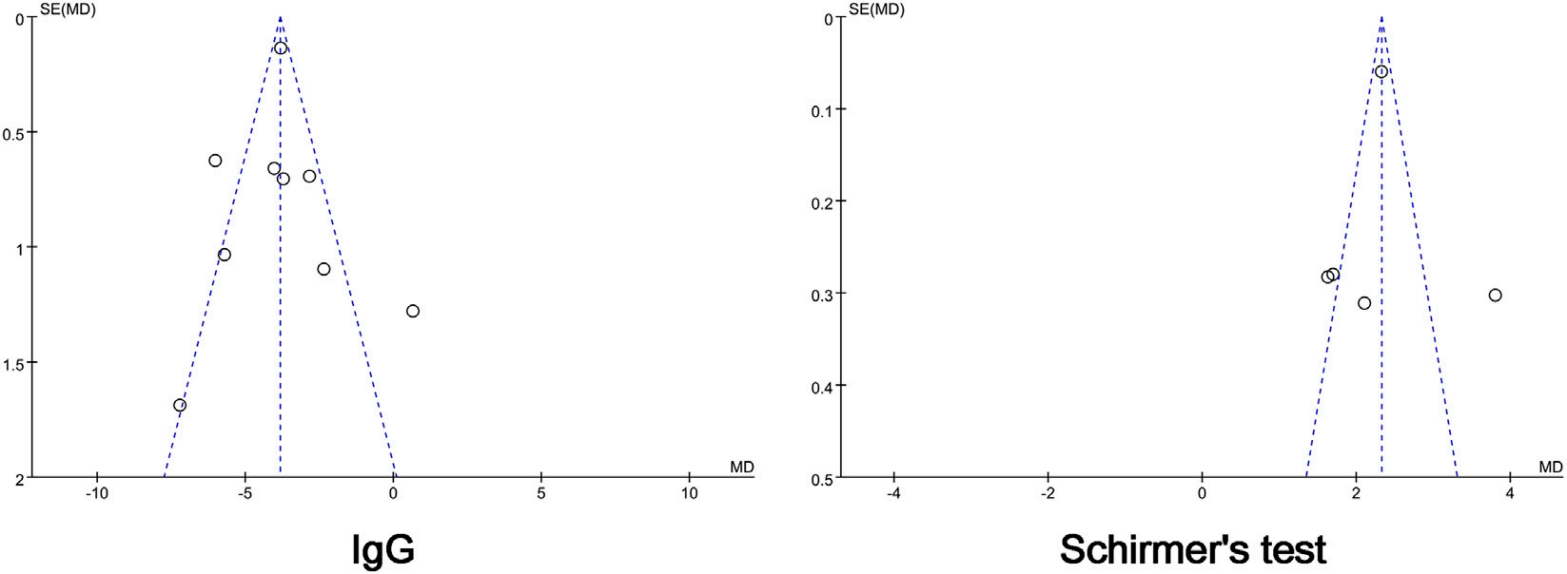
Funnel plots of comparison: IGU + HCQ + GC vs. HCQ + GC, outcome: IgG, Schirmer’s test.

Ten studies comparing IGU + HCQ + GC with HCQ + GC showed significant differences in ESR (MD = -5.18, 95% CI: -6.95 ∼ -3.41, *I* ^*2*^ = 0%, *p* < 0.00001), RF (MD = -5.79, 95% CI: -7.77 ∼ -3.80, *I* ^*2*^ = 0%, *p* < 0.00001), ESSPRI (MD = -2.03, 95% CI: -2.11 ∼ -1.94, *I* ^*2*^ = 39%, *p* < 0.00001), and efficacy rate (OR = 3.87, 95% CI: 2.44 ∼ 6.15, *I* ^*2*^ = 0%, *p* < 0.00001) (Figure 4). Heterogeneities in PLT count, salivary flow rate, and ESSDAI were eliminated after excluding one study, and significant differences were observed in these parameters. Next, funnel plots comparing IgG level and Schirmer’s test result were analyzed, and no publication bias was found (Figure 5).

#### IGU + GC vs. HCQ + GC

**FIGURE 6:**
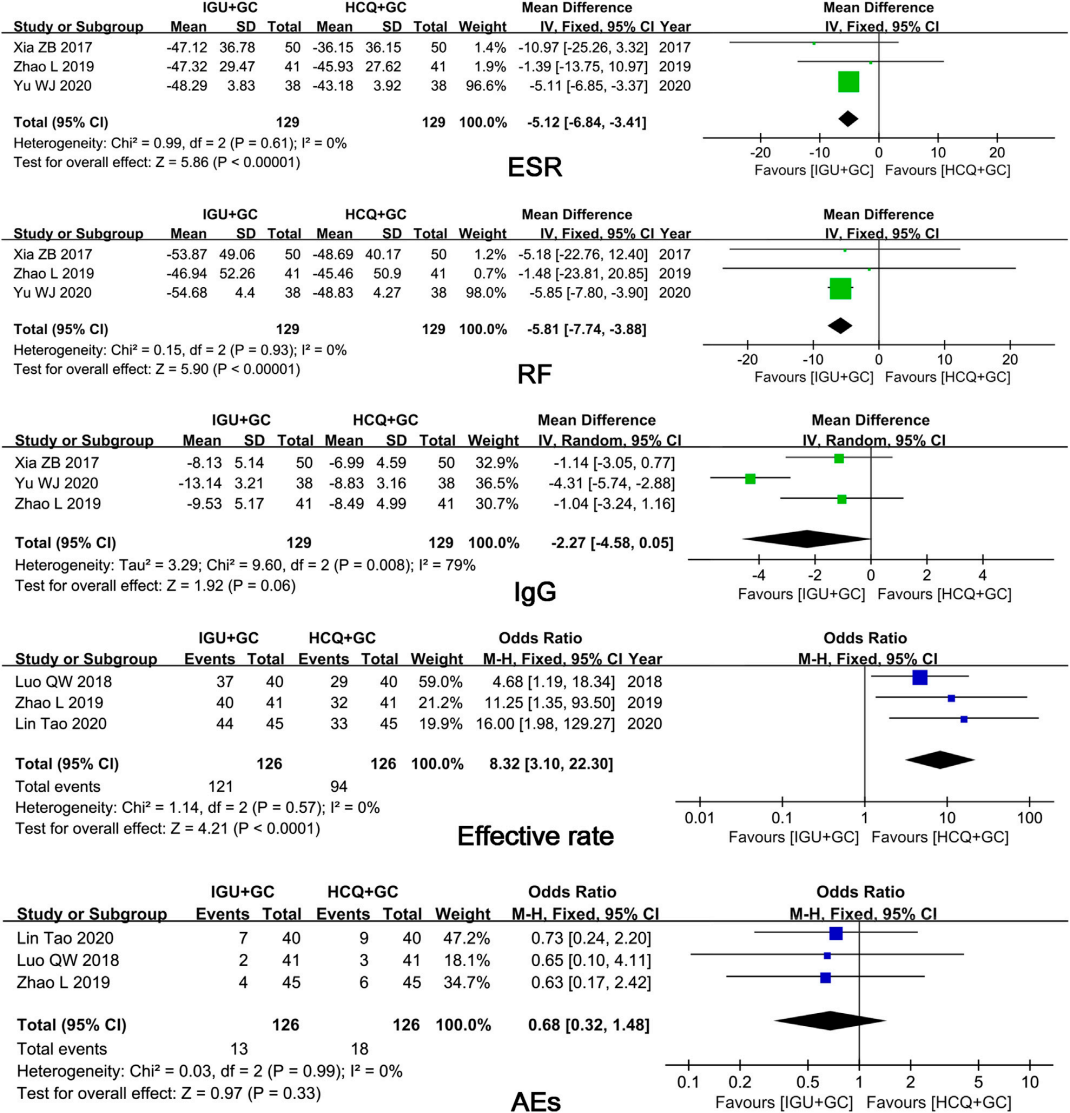
Forest plots of comparison: IGU + GC vs. HCQ + GC, outcome: ESR, RF, IgG, efficacy rate, AEs.

In six trials that compared IGU + GC with HCQ + GC, the pooled results showed significant differences in ESR, RF level, and efficacy rates (*p* ≤ 0.01) and in IgG level (*p* = 0.06) (Figure 6). However, only three studies and the excessive weightage of Yu’s study found the result of RF and ESR unconvincing. More studies are required.

#### IGU + HCQ + TGP vs. HCQ + TGP

**FIGURE 7:**
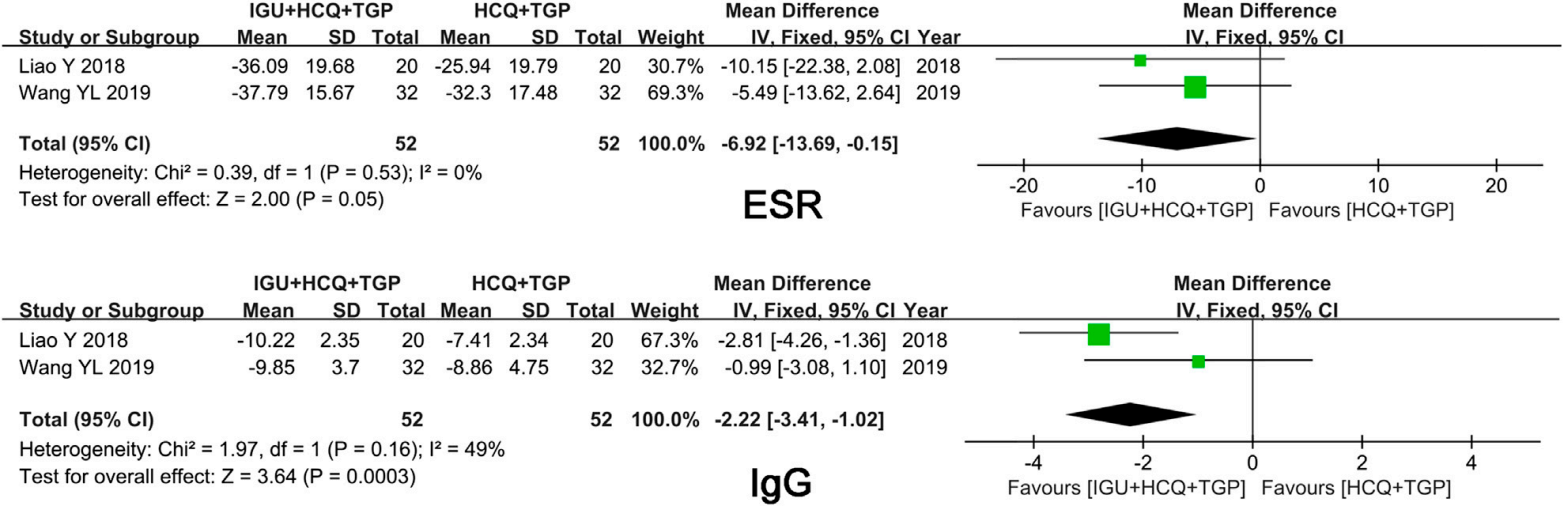
Forest plots of comparison: IGU + HCQ + TGP vs. HCQ + TG, outcome: ESR, IgG.

Pooled results from two trials comparing IGU + HCQ + TGP with HCQ + TGP showed significant differences in IgG level (*p* ≤ 0.01) and ESR (*p* = 0.05) (Figure 7).

#### IGU + HCQ + GC vs. LEF + HCQ + GC

Only one included trial compared IGU + HCQ + GC with LEF + HCQ + GC. Pooled results showed significant improvements in ESR, RF, and IgG levels, ESSDAI, and ESSPRI in the IGU group compared to those in the LEF group. However, data from only one trial cannot be considered convincing evidence; therefore, more RCT trials are needed.

### Safety of Interventions

#### IGU + HCQ + GC vs. HCQ + GC

AEs were reported in seven trials. No significant difference in the occurrence of AEs (*p* = 0.15) was found between IGU + HCQ + GC and HCQ + GC. Seventeen cases of gastrointestinal discomfort, five cases of rashes, seven cases of pruritus, five cases of abnormal liver function, three cases of leukopenia, and no cases of severe AEs were observed in 336 patients treated with IGU in seven trials.

#### IGU + GC vs. HCQ + GC

Three trials reported AEs. No significant differences were observed between the IGU + GC group and the HCQ + GC group (*p* = 0.33). Four cases of gastrointestinal discomfort, three cases of rashes, two cases of pruritus, two cases of abnormal liver function, and two cases of leukopenia were reported in the IGU group, consisting of 336 patients without severe AEs.

#### IGU + HCQ + TGP vs. HCQ + TGP

Only one included trial showed no significant difference in AEs between the IGU group and the non-IGU group. More trials are needed to derive a convincing conclusion.

#### IGU + HCQ + GC vs. LEF + HCQ + GC

Only one included trial showed lower risk of AEs in the IGU group than in the LEF group. However, data from one trial cannot be considered convincing evidence; therefore, more such trials are needed.

## Discussion

The present meta-analysis included 19 RCTs comprising 1,384 patients that compared IGU treatment with non-IGU treatment. Sensitivity analysis for processing heterogeneity was conducted to give our study a comprehensive version. Treatment duration in all the included studies was 12 weeks; thus, more time-dependent studies are needed to clarify whether the effectiveness and safety of IGU are time-dependent.

This meta-analysis suggested that IGU shows unique effects in improving the secretion function of the lacrimal and salivary glands when it was used in combination with HCQ + GC. However, high heterogeneity might be a problem. Sensitivity analysis showed that high heterogeneity in Schirmer’s test result was due to the two studies of Jiang W in 2014 and 2020, and high heterogeneity in salivary flow rate was due to the study of Xu et al. (2017). These high heterogeneities might originate from different equipment and operators. The superiority of IGU in improving eye dryness and mouth dryness might result from its inhibition of pro-inflammatory lymphocytes and immunoglobulin. IGU is generally considered as an anti-inflammatory and immunoregulatory drug that can improve symptoms and disease activities of patients with pSS by regulating the subpopulation of B cells, reducing the production of immunoglobulin, and inhibiting the proliferation of pro-inflammatory T cells and their influence on tissues (Jiang H. et al., 2020). BAFF (B cells activation factors), secreted by the secretion glands of pSS patients, binds to three different cell surface receptors: the BAFF receptor (BAFF-R), B cell maturation antigen (BCMA), and transmembrane activator and calcium modulator and cyclophilin ligand interactor (TACI). The BAFF–BCMA/TACI pathway promotes plasma cell survival and plays a role in humoral immunity (Jiang et al., 2011; Ou et al., 2012). While IGU was able to reduce both the BAFF level and plasma cell proportion by the BAFF–BCMA/TACI pathway, and thus decrease the production of IgG and prevent autoantibody-mediated damage, which was proven in Shao’s study (Shao et al., 2020). IGU was reported to be able to decrease the proportion of Th1 and Th17 cells and increase the proportion of Treg cells in patients with rheumatoid arthritis (RA) (Xu et al., 2015), which might also happen in pSS patients and reduce damage to the salivary glands.

Our meta-analysis suggested that the IGU group showed lower ESR than the non-IGU group, which was also observed in Shao’s study of 24 weeks (Shao et al., 2020). Previous research studies have shown that HCQ or TGP can reduce ESR (Feng et al., 2019; Bodewes et al., 2020); thus, IGU might enhance the anti-inflammatory activity of HCQ or TGP through a synergistic effect or independent pathways. In patients treated with IGU along with HCQ and GC, the RF level significantly decreased compared to that in patients treated with HCQ and GC. Although the superior efficacy of IGU in ESR and RF was shown in the comparison of IGU + GC vs. HCQ + GC, it was hard to draw conclusions for the high weightage of Yu WJ’s study (Yu, 2020) and three other studies. The pooled data indicated that the IgG level was significantly improved in the IGU group compared to that in the non-IGU group. However, no significant differences were observed between the IGU and HCQ groups. Previous studies have shown that HCQ significantly reduced the level of immunoglobulins in patients with pSS (Bodewes et al., 2020), thereby suggesting the possible synergistic effect of IGU with HCQ to reduce immunoglobulins and promote immune regulation. ESSPRI and ESSDAI, two indicators for assessing symptom perception and disease activity, have been widely used as endpoints in therapeutic studies of pSS (Seror et al., 2016). The improvement of ESR, RF, IgG, and exocrine glands influenced ESSDAI and ESSPRI. The trials included in this study for comparing IGU + HCQ + GC with HCQ + GC showed significant differences in ESSPRI and ESSDAI, which supported the use of the combination of IGU and other ISs for treating pSS.

In the safety assessment, no significant differences were found in AEs in the comparison of IGU with non-IGU or HCQ. Gastrointestinal discomfort was the most common AE in the IGU group. Severe AEs were not observed, suggesting that the inclusion of IGU in the treatment regimen did not increase the safety risk of patients with pSS. Shao’s trial also proved the safety of IGU, as no severe AEs were recorded in 24 weeks of treatment (Shao et al., 2020).

The present review has several limitations. First, all the included trials were conducted in China. While the trials of Japan or other countries were not found, and thus, the outcomes might not be representative of the global population. Second, some deficiencies were observed in the quality assessment of the methodology of the included studies: a high risk of bias in the blinding of participants and personnel and outcome evaluation was observed in all trials (100%). The randomization method was mentioned without any details in six studies (31.6%), and the allocation concealment method was not reported in six studies (31.6%). Third, a random-effect model was used to analyze outcomes with high heterogeneity, which might be due to different doses and different combinations of medicine in different trials. Fourth, in all trials, IGU at a dose of 25 mg once or twice a day was administered for 12 weeks; consequently, long-term outcome reports and whether the effectiveness and safety of IGU are dose- or time-dependent remain unclear. Although the study of Shao (Shao et al., 2020) provided us with more information on the effectiveness of IGU alone and on the safety of IGU in a longer therapeutic period, more data are required to derive valid conclusions. The results of this meta-analysis should therefore be interpreted with caution by taking these limitations into consideration. Hence, high-quality, multicenter, long-term RCTs are required to further assess the effectiveness and safety of IGU in treating patients with pSS.

## Conclusion

Our results suggest that IGU can significantly improve the clinical symptoms of patients with pSS, including inflammatory indices (ESR and IgG and RF levels), secretion function of the salivary and lacrimal glands (salivary flow rate and Schirmer’s test result), ESSDAI, and ESSPRI, when co-administered with IS therapy. The incidences of AEs in the IGU group were not higher than the non-IGU group. Gastrointestinal discomfort was the most common AE in the IGU group, but severe AEs were not reported. Therefore, IGU could be considered as a potentially useful and safe drug for treating pSS and could be used to enhance the immunoregulatory effects of ISs and to improve the lacrimal gland function in patients with pSS. Because of limitations of the studies included in this review, multicenter and long-term RCTs are needed to make a more comprehensive and integrated assessment of the safety and effectiveness of IGU for treating pSS.

## Data Availability

The original contributions presented in the study are included in the article/Supplementary Material, further inquiries can be directed to the corresponding author.
